# Integration of palliative rehabilitation in cancer care: a multinational mixed method study

**DOI:** 10.1186/s12904-024-01586-1

**Published:** 2024-11-18

**Authors:** Guro Birgitte Stene, May Aasebø Hauken, Hilde Hjelmeland Ahmedzai, Constance Gaard Storvestre, Skjalg Eirik Vervik, Joanne Bayly, Augusto Tommaso Caraceni, Stefania Costi, Guillaume Economos, Mai-Britt Guldin, Barry J. A. Laird, Lise Nottelmann, Matthew Maddocks, Andrew Toby Prevost, Julia Romeyer, Line Merethe Oldervoll

**Affiliations:** 1 Centre for Crisis Psychology, University of Bergen, Bergen, Norway; 2 Department of Neuromedicine and Movement Science, Faculty of Medicine and Health Sciences, Norwegian University of Science and Technology, Trondheim, Norway; 3 Cicely Saunders Institute of Palliative Care, Policy & Rehabilitation, Faculty of Nursing, Midwifery & Palliative Care, King’s College London, London, UK; 4 Fondazione IRCCS Instituto Nazionale dei Tumori, Milano, Italy; 5 Azienda USL – IRCCS of Reggio Emilia, Modena, Italy; 6 University of Modena and Reggio Emilia, Modena, Italy; 7 Centre Hospitalier Hôpital Lyon-Sud, Palliative Care Centre, Centre de soins palliatifs, Pierre-Benite, Lyon, France; 8 Research Unit for General Practice, Aarhus University, Aarhus, Denmark; 9 Western General Hospital and Institute of Genetics and Cancer, University of Edinburgh, Edinburgh, UK; 10 Research Unit, Department of Palliative Medicine, Bispebjerg and Frederiksberg Hospital, Copenhagen, Denmark; 11 Nightingale–Saunders Clinical Trials and Epidemiology Unit, Cicely Saunders Institute of Palliative Care, Policy & Rehabilitation, King’s College London and Nightingale-Saunders Clinical Trials and Epidemiology Unit, King’s College London, London, UK; 12 Hôpital Lyon Sud, Unitéde Soins Palliatifs, Pierre Bénite, Lyon, France; 13 The National Institute on Intellectual Disability and Community, Department of Mental Health, Faculty of Medicine and Health Sciences, Norwegian University of Science and Technology, Trondheim, Norway

**Keywords:** Palliative rehabilitation, Cancer, Disability, Integration, Health -service comparison, Mixed-method

## Abstract

**Background:**

Incurable cancer is a major contributor to societal suffering and disability, and palliative rehabilitation is recommended to be integrated within and between cancer services at all healthcare levels. However, little knowledge exists on how integration of palliative rehabilitation in cancer is understood and achieved in clinical practice. INSPIRE (Integrated short-term palliative rehabilitation to improve quality of life and equitable care access in incurable cancer) is a large European-funded project that aims to promote quality of life through a novel rehabilitation model for people disabled by advanced cancer.

**Aim:**

To compare the existing integration of palliative rehabilitation in cancer within official documents and in clinical practice across five European countries including United Kingdom, France, Denmark, Norway, and Italy.

**Methods:**

Mixed methods study with a concurrent research design, comprising a document analysis (*N* = 23), stakeholder interviews (*N* = 22), and an online survey (*N* = 225). Data from each sub-study were analysed separately before results were merged.

**Results:**

There was limited integration of palliative rehabilitation in cancer in official documents and in clinical practice, though some indicators of integration, including participation in multidisciplinary teams and adherence to standardised pathways, were identified in the survey. Notably, integration of palliative rehabilitation in cancer in clinical practice was observed within limited organisations in secondary healthcare systems, without widespread adoption. Although palliative rehabilitation in cancer as a concept was sparingly used by stakeholders, they recognised the need for a comprehensive approach including multidisciplinary teams that aligns with the individual patient’s needs and goals. Moreover, the ambiguous distinction between the terms ‘palliative rehabilitation’ and ‘palliative care’, insufficient funding, lack of well-defined care pathways and competence gaps among healthcare professionals represented barriers to integration of palliative rehabilitation in cancer into clinical practice.

**Conclusion:**

Integration of palliative rehabilitation in cancer was limited in the five EU partnership countries investigated. Clarifying the concept of palliative rehabilitation, including adoption of the concept into official documents and delineating it from palliative care, is essential for more successful integration. This can possibly be achieved by addressing the barriers identified and fostering close collaboration across disciplines.

**Supplementary Information:**

The online version contains supplementary material available at 10.1186/s12904-024-01586-1.

## Background

In Europe, 2.7 million people were diagnosed with cancer in 2020. Estimates suggest a 24% increase in cancer cases by 2034 making cancer the leading cause of death [1]. Simultaneously, advances in cancer treatment have resulted in improved life expectancy and survival for people with cancer, even those with incurable disease [2]. Living longer with incurable cancer comes with extended periods of burdensome symptoms, those affected are often older and have comorbidities, and suffer from loss of function (disability), one of the most critical and growing unmet care needs by patients [3]. Thus, there is a current and critical need for supportive and palliative healthcare services across Europe to meet the needs of a growing population of older, functionally impaired people living with incurable cancer.

To address this, access to specialised rehabilitation services aligned with what is regarded as traditional palliative care should be an essential component of supportive and palliative healthcare for all patients with incurable diseases [4, 5]. This involve offering patients a comprehensive approach, palliative care, that combines the goals of palliative care - addressing symptoms and the unique needs of people living with incurable cancer, and rehabilitation - with a specific focus on optimising function and enablement in their physical, psychological and social environments [6–8]. As a concept, palliative rehabilitation has evolved over some decades, leading to the current understanding of it as a function-directed care addressing the unique needs of patients with incurable cancers and their caregivers to improve quality of life [9–11].

In 2023, the World Health Organisation launched in 2023 a policy brief [12] where they define palliative rehabilitation “as the process of helping individuals with a progressive, often advanced or incurable disease reach their physical, psychological, and social potential consistent with physiological and environmental limitations and life preferences.” In accordance with this brief, palliative rehabilitation empowers people with incurable health conditions to actively manage their condition, reduce symptoms, and maintain independence and social engagement [13]. This approach allows people to enjoy the best possible quality of life, including towards the end of life [14]. Moreover, the potential benefits of improving integration are better access, enhanced quality, and greater efficiency in healthcare delivery [12]. Furthermore, palliative rehabilitation in patients with incurable cancer has recently become the subject of clinical trials assessing its feasibility [15] and effectiveness [16, 17]. Although these studies demonstrate some improvements in symptom- and functional outcomes, they are typically conducted across diverse care settings [18]. Further research on palliative rehabilitation is warranted to optimise interventions, access and delivery of care.

Consequently, a multinational consortium including seven European Union countries was established with the ambition to test an innovative model of palliative rehabilitation – INSPIRE (Integrated short-term palliative rehabilitation to improve quality of life and equitable care access in incurable cancer) - that can be integrated into routine care for people with incurable cancer — (Project no. 101057043 – INSPIRE). The overall framework of the INSPIRE project has been published in 2023 [19], and additional information about the INSPIRE project can be accessed on the consortium website [20].

Although the rationale for palliative rehabilitation in cancer appears logical, there is a clear need for further knowledge and understanding of how it can be understood and implemented across health care services in different European countries. As such, it was deemed crucial to the initiation of the INSPIRE project to ascertain the current integration of palliative rehabilitation within official documents and in clinical practice. This will serve as a foundational step for the INSPIRE clinical trial. The overall aim of this study was therefore to compare the current integration of palliative rehabilitation in cancer within the INSPIRE partnership countries. Specifically, we aimed to answer the following research questions:

1. How is palliative rehabilitation described in official documents?
2. How do stakeholders experience palliative rehabilitation in clinical practice?
3. How are indicators of integration of palliative rehabilitation reported in clinical practice?

## Methods

### Design

To address the three research questions, we employed a mixed methods approach with a concurrent research design, combining both quantitative and qualitative methods. Within this design quantitative and qualitative data are combined to obtain a more complete understanding of the phenomenon under study [21]. We incorporated three sub-studies: (1) a document analysis to gain insights, (2) interviews to explore experiences from stakeholders, and (3) an online survey to assess current clinical practice. We collected and analysed data from each sub-study independently, before synthesising the results to achieve a comprehensive and nuanced understanding [21].

### Eligibility criteria and recruitment

The following eligibility criteria were established for each of the three sub-studies:

*Document analyses*: official documents of relevance to cancer or palliative care services published by policy makers, charities or non-governmental organizations (NGOs); official policies or policy directives, strategies, official statements, and declarations, guidelines, reports, or white papers, (directed at a national level within the partnership countries), and as current publication as possible and/or updated versions.

*Stakeholder interviews*: For each country, we gathered data from a minimum of four stakeholders representing the following departments at each INSPIRE sites: primary healthcare, specialist healthcare (such as oncology department, palliative care unit, hospice, or rehabilitation service), and from a non-governmental organization (NGO) representing cancer patients/careers, with sufficient proficiency in verbal English to participate.

*Online survey*: Data collection for the survey was anonymous, and recruitment was facilitated through INSPIRE collaborators. An email with a link to the online survey was sent to all lead investigators. They forwarded the survey invitation to medical and nursing staff, physiotherapists, occupational therapists, psychologists, dietitian/nutritionists, pharmacists, and others who work with patients with incurable cancer at the trial sites.

### Data collection and analysis

*Document analysis*: We followed a four-step READ approach to document analysis in health policy research [22]. In step one (‘Ready your materials’), the lead investigators in each partnership country were instructed to identify 3–6 relevant documents based on the predefined eligibility criteria. Lead investigators were well acquainted with the INSPIRE project, considered experts within palliative rehabilitation in cancer and familiar with the national official documents in their respective countries. All identified documents were reviewed by the first author (GBS) and through a “back and forth” approach to ensure that eligible and relevant documents were included from each country, the final number of included documents were validated. In step two (Extract data), the lead investigators research teams (1–2 native language reviewers) read the documents thoroughly and extracted information using a pre-defined Excel Extraction Form (Supplementary file 1). In addition to extracting descriptive data about the documents (e.g. year of publication, type of document), the reviewers were instructed to extract data (e.g. words, sentences or paragraphs from the material) that specifically focused on rehabilitation (or a relevant term) within the context of incurable cancer or palliative care (for details, see Read Me in Supplementary file 1). To help the reviewers describe context and to guide analysis, four pre-defined topics were defined; population (type or stage of cancer), setting (primary or specialist level), timing (point in disease trajectory), and intervention (type and professionals involved). In step three (Analyse data), the researcher (GBS) converted the reviewer’s extractions into individual transcripts for each document and imported these into NVivo (Version 14, Lumiverio, USA). Independently, two researchers (GBS and LMO) read the transcripts for each document and sorted the data according to the pre-defined extraction topics. Any reflections made by the researchers were written down and used to support the synthesis process. In step four (Distil your findings), a synthesis process began with GBS and LMO individually merging the sorted data from each country, and then met to discuss the findings from each country. In this process, the data and any reflections made in the individual review and synthesis process was discussed and from this, the pre-defined extraction topics were refined into new themes for the final synthesis. Finally, each work package leader provided feedback on the synthesized findings from their respective country. Following this feedback, the two researchers collaboratively arrived at a conclusive synthesis across all documents and countries.

*Stakeholder interviews*: Stakeholder interviews were conducted by three senior researchers (MAH, HHA and LMO), all of whom have extensive experience in palliative, rehabilitation, and qualitative research. They are also part of the Norwegian consortium in the INSPIRE project. Out of the 22 interviews, three were conducted face to face, while the remaining took place digitally using platforms, e.g. Zoom or Microsoft Teams. Three interviews were conducted in Norwegian (two Norwegians and one Danish), while the remaining interviews were conducted in English. All interviewers were fluent in English, and all stakeholders were also proficient in English. Each interview followed a semi-structured interview guide, based on the study protocol and the definition of palliative rehabilitation (Supplementary file 2). The central question was: “*Can you please describe how palliative cancer rehabilitation is integrated in clinical practice in your country?*” Participants were encouraged to share their experiences and perspectives freely. Subsequent questions inquired into definition of palliative rehabilitation, its status as an essential service, access, and optimal delivery methods for the future Interviews lasted between 30 and 60 min, during which audio recordings were made and subsequently transcribed verbatim. To safeguard participants’ anonymity, no names or background information were included in the transcripts. Transcribed data were analysed using comparative qualitative analyses [23]. Initially, all interviews were individually read by two researchers (MAH and HHA) separately to identify meaningful units/ descriptive codes. These codes were based on the transcribed text and reflected the stakeholders’ own words, ensuring an ‘in vivo’ perspective. Subsequently, a discussion between the two researchers ensued until consensus was reached on 13 codes. Next, interviews from each partnership country were coded separately into the software Nvivo by MAH. Each country provided data that supported each code, except for the code ‘referral’ in Norway and France. Following this, the researchers independently summarized the extracted codes for each stakeholder in each country. Next, the researchers compared the content of the codes across stakeholders and engaged in further discussions to achieve consensus. A common content for each code in each country was extracted. Finally, the researchers collaboratively synthesized the findings in main themes and subthemes. The themes were validated by incorporating relevant quotes from the transcribed interviews.

*Online survey*: Data collection for the survey was conducted via a self-report online questionnaire, utilizing the digital platform Survey XACT (Rambøll, Denmark) (Supplementary file 3). The survey was based on the following key indicators of integration identified in the literature: (I) working in multidisciplinary teams, (II) following standardized care pathways, (III) having shared patient records between departments and levels of care, (IV) having joint multidisciplinary educational activities, (V) conducting routine needs assessments, and (VI) continuity of care between specialist and primary care levels [6, 24–27]. The questionnaire comprised 23 questions, including three background questions and 20 questions addressing palliative rehabilitation in cancer and the above indicators of integration. The type of questions included single-response, multiple-response, Likert scale, rating scale, and open-ended questions. The survey was pilot tested with eight individuals of whom six were health care professionals familiar with the fields of oncology, palliative care and rehabilitation. Two were psychology students who had no experience or expertise in these areas of health care.

Participant characteristics (country, professional background, and department/workplace) were summarized using descriptive statistics and reported as frequencies/percentages. Cross-tabulations were employed to analyse data by country and results tabulated using frequencies/percentages. The survey data was analysed using the Statistical Package for Social Sciences (SPSS IBM Version 28).

### Synthesis of the results

In the analysis of a concurrent mixed-method design, the final step involved identifying content that is represented in all three data sets and comparing, contrasting, and synthesizing the results [21]. Data from each sub-study were extracted and consolidated into a common display. Subsequently, we examined convergences and divergences to generate a cohesive and compete synthesis of the results.

## Results

### Participants

Characteristics of the official documents, stakeholders and survey participants are outlined in Table 1. A total of 23 documents were identified from the partnership countries. These primarily consisted of national strategies, guidelines or policies published in the period 2004–2023, with the majority published after 2015. A total of 22 stakeholders were interviewed between November 20th, 2022, and February 9th, 2023. The online survey was conducted between May 1st to July 31st, 2023, with a total of 225 respondents.

**Table 1:** Total numbers and numbers per country for the three sub-studies

Variable	Document analyses		Stakeholder interviews		Survey				
	n	%	n	%	n		%		
Total documents/participants	23	100	22	100	225		100		
Country									
Denmark	4	17.4	5	22.7	5		2.2		
France	6	26.1	3	14.3	20		8.9		
Italy	4	17.4	4	18.1	140		62.9		
Norway	4	17.4	4	18.1	17		7.6		
UK	5	21.7	6	27.3	43		19.1		

### Document analyses

A brief overview of all the included official documents is provided in Table 2. Twelve out of 23 official documents (48%) described rehabilitation in the context of palliative care; 4 out of 4 (100%) in Italy, 3 out of 4 (75%) in Denmark and Norway, 2 out of 4 (50%) in England and Scotland, and 2 out of 6 (33%) in France.

**Table 2:** Overview of publisher (year), type of document, objective, and status for integration of rehabilitation in the context of palliative care in the official documents included in the analysis (*n* = 23)

Publisher (year)	Type of document	Objective	Status
**Denmark**			
Danish Health Authority (2018)	Policy	To outline the responsibilities of the municipalities regarding rehabilitation.	No
Danish Health Authority (2018)	Care pathway	To describe recommended care pathways for rehabilitation and palliative care for cancer.	Yes
Rehabilitation Forum Denmark (2022)	White paper	To provide rehabilitation services and current challenges provided by and used by all stakeholders.	Yes
Danish Cancer Society (2021)	Report	To monitor the quality of rehabilitation services provided to cancer patients.	Yes
**France**			
Haute Autorité de Sant (2002)	Guideline	These guidelines aim to guarantee the right to and access to palliative care.	Yes
Direction Générale d*’* l’Offre de Soins (2023)	Strategy	This document is a policy guiding the further development and structuration of the palliative care offer across the state between 2024 and 2034.	Yes
Association française des Soins Oncologiques de Support (2013, 2018)	Guideline	To promote physical activity and rehabilitation in breast cancers all along the disease trajectory.	No
National Cancer Institute (2021)	Guideline	To define at a national level the organizational principles related to the implementation of the support care pathway for patients with cancer.	No
Haute Autorité de Santé (2019)	Guideline	To guide the promotion, consultation and prescription of physical activity and sport for health, including specific guidance for people with three most common cancers (breast, colon and prostate).	No
National Cancer Institute (2017)	Guideline	To summarize the data on physical activity benefits in cancer and provide guidelines on the integration of physical activity during and after cancer treatments.	No
**Italy**			
Italian Association of Medical Oncology/ Italian Society of Palliative Care (2015)	Other (consensus)	To guide healthcare professionals to offer the most appropriate treatment pathway for patients with advanced cancer.	Yes
Permanent Conference for relations between the State, the Regions and the autonomous Provinces of Trento and Bolzano (2019)	Policy Directive	To revise the organizational guidelines and recommendations for the Oncology Network that integrates acute and post-acute hospital activity for the promotion and improvement of the quality, safety and appropriateness of supportive interventions.	Yes
Italian Ministry of Health (2019)	Official statement or declaration	To summaries the goals achieved and the critical issues that have emerged in the process of implementation and development of assistance networks in palliative care and pain therapy for adults and children, eight years after the entry into force of Law No. 38 of 15 March 2010.	Yes
Italian Ministry of Health (2021)	Policy	To ensure appropriate qualified palliative care and pain therapy for patient and his family for the period 2010–2020.	Yes
**Norway**			
Ministry of Health and Care Services in Norway (2018)	Strategy	To provide directives for cancer care in Norway for the period 2018–2022.	Yes
Norwegian Cancer Society (2018)	Strategy	To work to prevent and fighting cancer as well as improving the quality of life for people with cancer and relatives.	No
Norwegian Directorate of Health (2019)	Guideline	To improve the treatment of patients with incurable cancers and a limited lifespan, and to ensure an equally good treatment offer throughout the country.	Yes
Norwegian Directorate of Health (2017)	Care Pathway	To ensure safe and proper conditions for those who have received a cancer diagnosis and that the need for follow-up beyond the cancer treatment itself is secured.	Yes
**England and Scotland**			
NHS England (2019)	Strategy	To detail what is required by the National health Service towards 2029.	No
NHS England (2018)	Guideline	To provide practical advice and case studies to support Allied Health Professionals in Action.	Yes
The Scottish Government (2008)	Strategy	To set out the specific challenges for cancer care in Scotland as well as strategies for improvement.	No
National Institute for Clinical Excellence (2004)	Guideline	To guide how to improve the quality of supportive and palliative care for cancer patients.	Yes
NHS England (2022)	Strategy	To provide strategic direction to AHPs across England in the period 2022–2027 and help AHPs maximize their contribution to improve health outcomes for all, provide better quality care, and improve sustainability of health and care services.	No

The final synthesis for each partner country is summarized in Table 3. During the synthesis process, five main themes emerged: Needs and access to rehabilitation; Settings, responsible parties, and essential services; Timing and care pathways; Goals, interventions and professionals, and Research and future palliative rehabilitation. Citations for all 23 included official documents, along with their individual synthesis, are available in Supplementary files 4.

**Table 3:** Main themes from extraction and analysis of documents (*n* = 12)

Main theme	Needs and access to rehabilitation	Settings, responsible parties and essential services	Timing and care pathways	Goals, interventions, and professionals	Research and future palliative rehabilitation
Denmark	People living with cancer in all phases of the disease trajectory, including those living with incurable disease.	Community health care	R and PC separate care pathways Time of referral inconsistent, but important for integration of R and PC.	R and PC overlap but goals are unclear. Defines the term “palliative rehabilitation”.	Advocates for integration of R and PC although differences still exist.
France	People who need palliative care services.	Hospitals	R is part of holistic care planning in PC	MDT should include rehabilitation practitioners.	ND
Italy	People with advanced cancer with complex needs and need of PC, also in terminal stage.	Both in the specialist- and community health care	Essential part of care pathway throughout the disease trajectory	Comprehensive approach with focus on QoL, R essential part of PC service, multidisciplinary team require RP competence.	ND
Norway	All people with cancer regardless of curative or palliative intention.	Both in hospital and community setting	Early in the disease trajectory	R considered part of PC through common goals and use of MDT including RPs.	R should be available to all cancer patients
UK	All with people with cancer, although access is variable but expanding for people with progressively deteriorating cancers.	Hospitals, hospices, and primary care	Throughout disease trajectory	R as part of supportive care, is essential to patients with complex needs to improve QoL. Assessment of needs should include R. AHPs have specialist role and part in the PC MDT.	Research on R in PC is lacking and should focus on the patients’ needs.

PR = Palliative Rehabilitation R = Rehabilitation; PC = Palliative Care, RPs = Rehabilitation Professionals, MDTs = Multidisciplinary Teams; ND = Not described, AHPs = Allied Health Professionals

### Stakeholder interviews

The synthesis of stakeholder interviews from each country is detailed in Supplementary file 5, while the overall findings are presented in Table 4 and described below.

**Table 4:** Overall findings from stakeholder interviews about palliative rehabilitation in cancer care

Main themes	Subthemes
1: Varied understanding and use of the term palliative rehabilitation in cancer	1a: Stakeholders expressed a common understanding of the main components in palliative rehabilitation in cancer.
	1b: Stakeholders experienced an unclear distinction between palliative rehabilitation in cancer and palliative care.
2: Consensus on intervention types and professionals involved in palliative rehabilitation	2a: Stakeholders agreed that palliative rehabilitation should be built on multidimensional interventions aligned with individual patient needs and goals.
	2b: Stakeholders agreed that palliative rehabilitation must embrace a multi-professional approach and collaborative efforts.
3: Limited integration of palliative rehabilitation in cancer in clinical practice	3a: Access to palliative rehabilitation was experienced as a postcode lottery.
	3b: Lack of pathways and funding issues was outlined as primary barriers to the integration of palliative rehabilitation in clinical practice.

### Main theme 1: Varied understanding and use of the term palliative rehabilitation

The analyses revealed that stakeholders within and across participating countries held diverse interpretation of the term. This main theme was further explored by two subthemes.

The first sub-theme, «Stakeholders expressed a common understanding of the main components in palliative rehabilitation in cancer”, showed that stakeholders often found the concept elusive and challenging:

*“I think it’s a concept that’s probably not very well defined in many settings. So*,* it varies across the country whether it’s community acute or even sub-acute rehabilitation”* (Stakeholder 2, UK).

Most participants did not use the concept in clinical practice nor were they able to define it. Still, most stakeholders, upon reflection, shared a mutual understanding of core components of WHO’s definition of palliative rehabilitation in cancer. Here, they viewed palliative rehabilitation in cancer as a holistic, goal-oriented approach for cancer patients with incurable and life limiting cancer. The overarching aim, according to stakeholders, was to enhance or maintain quality of life and function based on individual patient needs and goals. This approach allows patients to live as actively and independently as possible within the constraints of their illness:

*“Palliative rehabilitation in cancer aims to improve the patient’s quality of life through a better everyday life*,* to support people in their everyday lives so they still are able to pursue their everyday tasks and to be as independent as possible with home tasks and so on”* (Stakeholder 4, Denmark”.)

The second subtheme “The stakeholders experienced an unclear distinction between palliative rehabilitation and palliative care”, highlighted the blurred boundary between palliative rehabilitation and palliative care. While stakeholders could identify the main components of palliative rehabilitation in cancer, they perceived it as operating within a ‘grey zone’ straddling rehabilitation and palliation. Although all stakeholders acknowledged its connection to palliative care, the integration of rehabilitation within this framework varied. Some stakeholders associated rehabilitation primarily with the early phase of the disease trajectory, others linked it more broadly to content related to holistic palliation but more than symptom relief, while some stakeholders stated it was the same as palliative care:

*«I think that rehabilitation is integrated in what we do in palliative care*,* without putting it into words. (…) For me*,* if you provide good palliative care*,* then it’s not only symptom relief*,* but a very strong element of optimalisation of function (…) to help patients to function optimal as long as possible»* (Stakeholder 4, Norway).

### Main theme 2: Consensus on intervention types and professionals involved in palliative rehabilitation

The second main theme focused on stakeholders’ perspectives regarding care interventions and professionals responsible for delivering them. This main theme was further elucidated by two sub themes. The first subtheme, «Stakeholders agreed that palliative rehabilitation should be built on multidimensional interventions aligned with individual patient needs and goals”, focused on the content of palliative rehabilitation interventions. Stakeholders unanimously recognized that palliative rehabilitation in cancer encompasses a range of multidimensional interventions tailored to each patient’s specific needs and preferences. Drawing from the holistic approach in palliative care, they emphasized that the elements of care should align with the patient’s unique circumstances:

*“The components of palliative rehabilitation can vary widely*,* but they must always be based on the patient’s needs and preferences (Stakeholder 2*,* Italy).*

Despite this consensus, there were variations in the specific interventions included. Notably, these differences did not correlate with country boundaries. Commonly reported interventions included physical exercise, nutrition, psychological support, psychoeducation (both individual and group based), and various assistive aids. Additionally, stakeholders highlighted the importance of addressing the patient’s home environment, symptom management, assistive technology, energy conserving, lifestyle modifications (such as smoking cessation and alcohol reduction), peer support, and complementary therapies.

While most interventions centred on individual patients, stakeholders acknowledged the crucial role of education and involving the patient’s carers. However, only a few stakeholders from Norway, Denmark, and Italy explicitly referred to interventions relating to the needs of family carers.

The second subtheme, “Stakeholders agreed that palliative rehabilitation must embrace a multi-professional approach and collaborative efforts”, emphasized the necessity of a multi-professional and interdisciplinary approach in the delivery of palliative rehabilitation in cancer. All stakeholders concurred that effective palliative rehabilitation in cancer requires collaboration among various professionals. These experts should work together seamlessly to address the patients’ holistic needs:

*“The multidisciplinary approach is essential. You cannot call it palliative rehabilitation if it’s not interdisciplinary”* (Stakeholder 1, Norway).

The most frequently mentioned key professionals in palliative care included doctors, (cancer)nurses, nutritionist/dietician, social workers, psychologists, physiotherapists, and occupational therapists. Some stakeholders noted that the two latter groups had rehabilitation more integrated in their education and practical experiences compared to other professionals, making them particularly crucial in care delivery. Additionally, a few stakeholders highlighted the involvement of professionals in complementary therapy and wellbeing assistance (in the UK), professionals from nurseries and schools, and social security services (in Norway). Furthermore, stakeholders from Norway and Denmark emphasized the role of chaplains, while those from Italy and UK mentioned speech therapists. Overall, there was unanimous agreement among stakeholders that professionals ideally collaborate in multidisciplinary teams to provide palliative rehabilitation in cancer. However, it is worth noting that teams providing palliative rehabilitation were primarily associated with hospitals in certain locations within Denmark and the UK.

### Main theme 3: Limited integration of palliative rehabilitation in cancer in clinical practice

The third main theme highlights the challenges related to the integration of palliative rehabilitation in clinical practice. It acknowledges that, in general, palliative rehabilitation was either inadequately or not integrated at all into palliative cancer care within hospital and primary healthcare settings. Interestingly, when discussing integration, most stakeholders tended to focus on the integration of palliative care rather than rehabilitation, reflecting the varied understanding of the term identified in theme 1. Supporting this view, dedicated integrated services exist only in a few hospital settings in Denmark and the UK. Overall, this was limited to isolated ‘silos’, understood as existing in separate units or organisations and lacking integrated interdisciplinary teams across different healthcare levels. Collaboration between professionals from various disciplines remains limited, with existing collaboration primarily centred around referrals or selected outreach services from hospitals to patients’ home:

*«We are still very much in the embryonic stage in the UK regarding that concept [palliative rehabilitation in cancer]. The government set that requirement in early 2020*,* but even in 2023 they still do not know at the strategy level how to deliver that integrated care. They’re still not working as a team. (….) The secondary care*,* the hospital care*,* and the primary care (…) they’re talking*,* but that’s all they’re doing. They’re not putting systems in place. (…) In some parts it’s slightly better*,* in some parts it isn’t”* (Stakeholder 6, UK).

This main theme is further elaborated by two sub-themes. The first subtheme, “Access to palliative rehabilitation was experienced as a postcode lottery”, showed that while all stakeholders recognized rehabilitation and palliative care as essential services, cancer patients’ access to such care remains limited both within and across countries. Interestingly, most stakeholders found that other patient groups, such as those with stroke or heart diseases, seem to have better access to rehabilitation services than palliative cancer patients. Geographic location plays a significant role, with access being more favourable in larger cities compared to rural areas. Consequently, several stakeholders described access as a ‘secret service’ or a ‘postcode lottery’:

*“I think many palliative rehabilitation services will argue that they do not have the capacity to see every single person (…). And even obviously we have some special teams in certain boroughs (…) but it’s a bit of a post code lottery”* (Stakeholder 2, UK).

The second subtheme, “Lack of pathways and funding issues was outlined as primary barriers to integration of palliative rehabilitation in clinical practice”, shows that most stakeholders reported that their countries lack specific pathways for cancer patients with incurable disease to access care. Instead, access often depends on chance encounter with dedicated professionals or referrals to the limited existing services:

*“If someone asks you where I find guidelines*,* pathways*,* they go to Macmillan ones. And basically*,* it’s just saying give them palliative care*,* and that’s rehabilitation”* (Stakeholder 1, UK).

While all stakeholders acknowledged the importance of integrated care, they also recognized that it remains a resource-constrained area. Dedicated funding is lacking, and its availability often depends on local priorities. Consequently, several stakeholders emphasized the critical need for funding to educate, develop, and sustain the much-needed professional expertise. Although most stakeholders agreed that palliative rehabilitation has not yet been fully integrated into clinical practice, they offered valuable suggestions for future development: First, stakeholders mentioned that establishing clear national priorities and allocating dedicated funding would enhance implementation. Then, they argued that developing standardized care pathways and guidelines specific to palliative rehabilitation in cancer would facilitate consistent and effective care delivery. Furthermore, implementing focused educational programs for healthcare professionals would enhance their competence in this complex area. Another important factor was integration from the moment of diagnosis that would ensure early intervention and comprehensive support. Then, regular assessments of patients’ need, considering physical, psychological, and social aspects, were viewed as essential. Embedding rehabilitation within existing palliative or supportive care services was suggested to ensure a holistic approach to patient well-being. Finally, building upon established structures, such as centres of excellence in palliative care, professional networks, individual care plans, specialist education, and dedicated rehabilitation centres, were suggested to strengthen implementation.

However, opinions on the optimal location for providing care varied among stakeholders within and across countries. Those working in primary healthcare settings recommended offering care near or within patients’ homes. In contrast, specialists primarily endorsed hospital-based care, emphasising its infrastructure and alignment with hospital-driven initiatives:

*I think that the best way to deliver palliative rehabilitation is to start delivering it in the oncology world (…) because patients are already in palliative care (…) this is the best time to provide them an intervention to help them manage their disease*,* be empowered. And after that*,* if the symptomatology change*,* if people change their priorities*,* then they can meet again the practitioner to redefine the objectives and the goals of this rehabilitation (Stakeholder 1*,* France).*

### Online survey

Most respondents (87%) reported being part of a multidisciplinary team (MDT) in their work with cancer patients. This was consistent across all five countries as indicated in Table 5. Regarding frequency of MDT meetings, 59% of respondents reported having weekly meetings, 12% reported having daily meetings, and 11% reported a monthly frequency of the MDT meetings. The five professions most frequently reported as members of MDTs were palliative medicine consultants/specialists (48.4%), oncologists (45.3%), psychologists (44%), palliative care nurses (41.3%) and physiotherapists (35%).

**Table 5:** Responses to indicators of integration

	Membership of MDT *N* = 215			Routine screening for rehabilitation needs *N* = 201			Multi professional educational activities *N* = 191			Standardised Care Pathways (SCP) *N* = 206			If SCP, is rehabilitation a component in the SCP *N* = 144		
Country	Yes n (%)	No n (%)	Different structure n (%)	Yes n (%)	No n (%)	Don’t know n (%)	Yes n (%)	No n (%)	Don’t know n (%)	Yes n (%)	No n (%)	Don’t know n (%)	Yes n (%)	No n (%)	Don’t know n (%)
Denmark	4 (80)	1 (20)	0	3 (75)	0	1 (25)	4 (100)	0	0	4 (80)	0 (0)	1 (20)	2 (67)	0	1 (33)
France	15 (94)	1 (6)	0	6 (43)	6 (43)	2 (14)	5 (39)	7 (54)	1 (8)	8 (57)	2 (14)	4 (29)	4 (50)	4 (50)	0
Italy	119 (88)	14 (10)	2 (2)	63 (49)	38 (30)	28 (22)	89 (73)	22 (18)	11 (9)	99 (76)	17 (13)	15 (11)	63 (64)	14 (14)	21 (21)
Norway	15 (88)	1 (6)	1 (6)	3 (19)	11 (69)	2 (13)	8 (53)	5 (33)	2 (13)	12 (75)	2 (12)	2 (13)	3 (25)	8 (67)	1 (8)
UK	35 (83)	4 (10)	3 (7)	14 (37)	16 (42)	8 (21)	21 (57)	15 (41)	1 (3)	24 (60)	13 (32)	3 (8)	6 (26)	8 (35)	9 (39)
Total	188 (87)	21 (10)	6 (3)	89 (44)	71 (35)	41 (20)	127 (67)	49 (26)	15 (8)	147 (71)	34 (17)	25 (12)	78 (54)	34 (24)	32 (22)

A key indicator for integrated healthcare relies on the utilization of standardized care pathways (SCPs) in follow-up of cancer patients (Table 5). Of the 147 participants (71%) who reported adhering to standardized care pathways, 78 (54%) stated that rehabilitation was a component within that pathway.

Regarding the indicator of shared patient records, 56% of respondents reported that electronic patient records were shared between all departments within the hospital. However, only 12% indicated that records were shared across all levels of care (primary, secondary, and tertiary). Overall, 56% reported that the applied IT platform functioned satisfactorily in terms of facilitating collaboration and integration among professions, departments, and levels of care. Still, there were significant variations between countries on satisfaction levels with the IT platform, ranging from 20% (Norway) to 85% (France) in individual countries.

A fourth indicator for integrated healthcare pertains to regular multiprofessional educational activities in the workplace. Considering all countries combined, 67% confirmed that this practice was in place. Again, there were substantial variations between the countries, as indicated in Table 5. Only 9% reported having joint educational activities between palliative care and rehabilitation services.

Relating to the question whether patients with incurable cancer were routinely assessed for rehabilitation needs in their workplace, 89 respondents (44%) confirmed that they were. However, substantial differences between countries were observed for this indicator too (Table 5). The 71 respondents (35%) who reported not routinely assessing patients with incurable cancer for rehabilitation needs were asked to identify other triggering factors for referral to palliative rehabilitation in this patient group. Poor physical functioning was most frequently reported (61%), followed by planned discharge of the patient from the hospital back to their home (39%). Requests from patients (31%) or their careers (24%) were also reported as triggering factors, whereas only 7 (10%) reported that referral to palliative rehabilitation was set out in patient pathways or guidelines for this group. 18 respondents (25%) reported that they did not refer patients with incurable cancer to palliative rehabilitation. When asked to rate on a scale from 0 (poorly) to 100 (excellent), the mean score for continuity of care between secondary and primary care was 67 for all countries combined.

Table 6 presents the respondents’ level of agreement on statements concerning palliative care and palliative rehabilitation. Forty-five respondents (25%) agreed or strongly agreed with statement 1, asserting that palliative care and palliative rehabilitation offer the same service. Thirty-one percentage could not decide whether to agree or disagree with this statement. Twenty six percentage agreed or strongly agreed with statement 4, expressing the view that rehabilitation is most appropriate for curative cancer patients. Further, on statement 5, “Rehabilitation is not appropriate for cancer patients towards the end of life”, 17% agreed or strongly agreed, whereas 61% disagreed or strongly disagreed with this statement.

**Table 6:** Respondents’ views on the following five statements (*N* = 184)

	Statement 1 Palliative care and palliative rehabilitation offer the same services			Statement 2 Palliative care focuses on symptom palliation			Statement 3 Palliative rehabilitation focuses on physical function			Statement 4 Rehabilitation is most appropriate for curative cancer patients			Statement 5 Rehabilitation is not appropriate for cancer patients towards the end of life			
**Country**	A/SA n (%)	NN n (%)	D/SD n (%)	A/SA n (%)	NN n (%)	D/SD n (%)	A/SA n (%)	NN n (%)	D/SD n (%)	A/SA n (%)	NN n (%)	D/SD n (%)	A/SA n (%)	NN n (%)	D/SD n (%)	
Denmark	0	2 (50)	2 (50)	1 (25)	0	3 (75)	2 (50)	0	2 (50)	0	0	4 (100)	1 (25)	0	3 (75)	
France	2 (17)	1 (8)	9 (75)	9 (75)	1 (8)	2 (17)	7 (58)	2 (17)	3 (25)	2 (17)	2 (17)	8 (67)	0	3 (25)	9 (75)	
Italy	34 (29)	36 (31)	47 (40)	79 (68)	9 (8)	29 (25)	56 (48)	27 (23)	34 (29)	37 (32)	29 (25)	51 (44)	23 (20)	26 (22)	68 (58)	
Norway	4 (29)	6 (43)	4 (29)	7 (50)	4 (29)	3 (21)	6 (43)	6 (43)	2 (14)	6 (43)	4 (29)	4 (29)	5 (36)	6 (43)	3 (21)	
UK	5 (14)	13 (35)	19 (51)	21 (57)	4 (11)	12 (32)	13 (35)	9 (24)	15 (41)	2 (5)	8 (22)	27 (73)	3 (8)	4 (11)	30 (81)	
Total	45 (25)	58 (31)	81 (44)	117 (64)	18 (10)	49 (27)	84 (46)	44 (24)	56 (30)	47 (26)	43 (23)	94 (51)	32 (17)	39 (21)	113 (61)	

A/SA = Agree/Strongly Agree; NN = Neither agree/Nor disagree/Don’t know; D/SD = Disagree/Strongly Disagree

### Synthesis of the results

The synthesis of the results is presented in Table 7.

**Table 7:** Synthesis of the combined results

Sub study	Synthesis
**Document analyses**	Integration of palliative rehabilitation in cancer is limited in official documents: • Rehabilitation was mentioned in the context of palliative care in 12 out of 23 documents (48%). • The concept of palliative rehabilitation in cancer appeared in one document (Denmark). • Rehabilitation and palliative care have separate care pathways, but there is some overlap in terms of goals, timing, organization, and professional content. • Allied health professionals and rehabilitation professionals are required in multidisciplinary teams to improve assessment of patients’ needs and access to rehabilitation in palliative care. • Research on palliative rehabilitation of cancer patients is lacking.
**Stakeholder interviews**	Limited integration of palliative rehabilitation in cancer in clinical practice: • There is a common understanding of the main components in the definition of palliative rehabilitation in cancer, but an unclear distinction between palliative rehabilitation and palliative care. • High agreement that palliative rehabilitation in cancer interventions is based on multidimensional approaches tailored to the patient’s needs and goals to enhance /uphold quality of life. • Access to palliative rehabilitation in cancer is described as a postcode lottery, with challenges related to funding, care pathways, and education.
**Survey**	Indicators of integration are present in clinical practice but limited for palliative rehabilitation in cancer: • Nearly all respondents work in multidisciplinary teams (87%) and follow standardised care pathways (SCPs) (71%) in follow-up of cancer patients. • Just over half of those following SCPs report that rehabilitation is a component of the applied standardized care pathways for cancer patients. • 25% of respondents believe that palliative care and palliative cancer rehabilitation offer the same services. • Nearly one fifth perceive that cancer rehabilitation is not appropriate towards the end of life, though 81% disagreed. • Routine assessment for rehabilitation needs in incurable cancer patients was reported by less than half of the respondents (44%). • Respondents rate continuity of care between healthcare levels relatively high with a score of 67 (0 = poorly to 100 = excellent).
**Synthesis of all sub studies**	The overall results highlight that integration of palliative rehabilitation in cancer remains limited within official documents and in clinical practice, despite the presence of some integration indicators. Palliative rehabilitation in cancer integration is primarily observed within specific organisations in secondary healthcare systems in Denmark and the UK. However, widespread adoption remains limited. Stakeholders recognize that effective palliative rehabilitation in cancer implementation should be multidimensional, and that interventions must align with individual patient needs and goals, emphasizing a holistic approach. An ambiguous distinction exists between palliative rehabilitation in cancer and existing palliative care. Insufficient funding, lack of palliative rehabilitation in cancer included in care pathways, and competence deficiencies among healthcare professionals represent barriers to integration of palliative rehabilitation in cancer into clinical practice.

## Discussion

The aim of this study is to compare the current integration of palliative rehabilitation in cancer within the INSPIRE partnership countries. The synthesis of findings from three sub-studies revealed that integration remains limited within all countries, despite the presence of some indicators of integration demonstrated in the survey. Key findings to be discussed include limited integration in official documents, scarce integration in clinical practice, holistic approach, palliative rehabilitation versus palliative care, and barriers to integration.

The scarcity of information about the integration of palliative rehabilitation within official documents across countries is a cause of concern. These types of documents (policies, strategies, and guidelines) play a pivotal role as valuable resources and roadmaps for healthcare professionals, policymakers, and funders. Consequently, such documents are crucial in resource allocation, strategic guidance for healthcare services, enhancing patient outcomes, guidance for healthcare professionals, promoting interdisciplinary collaboration, and quality assurance [12, 28]. Notably, only one Danish official document acknowledged the term palliative rehabilitation in cancer. The interviews revealed that palliative rehabilitation in cancer was primarily observed within limited organisations in secondary healthcare systems in Denmark and the UK without a widespread adoption. These findings align with the WHO’s policy brief, identifying cases of palliative rehabilitation in cancer with examples from Denmark, UK, and Italy [12], while we could not identify integration of palliative rehabilitation in cancer in Italy. The paucity of text in official documents highlights the need for a broader recognition and adoption of palliative rehabilitation in cancer both in official documents and clinical practice. The recent WHO policy brief is therefore crucial to this agenda [12].

A sparse level of integration was also evident in the stakeholder interviews, indicating that care was primarily provided in isolated ‘silos’ and lacking integrated multidisciplinary teams across different healthcare levels. In contrast, the survey revealed a more positive picture relating to being part of multidisciplinary teams, shared patient records, using standardized care pathways for cancer including palliative rehabilitation, and collaboration between healthcare services.

In the stakeholder interviews, it became evident that defining palliative rehabilitation in cancer posed challenges. However, across the sub-studies, a consensus emerged that care should be constructed with a holistic approach, considering emotional well-being, mental health, and social context alongside physical health. These findings align with prior literature, which also underscores the importance of a holistic perspective in cancer care [6, 7, 12]. Furthermore, the results highlight a strong agreement that care must be multidimensional and tailored to individual patient needs and goals, calling for multidisciplinary interventions and collaboration across disciplines. In line with previous research, palliative rehabilitation extends beyond physical exercise interventions and focuses on supporting patients’ overall function and quality of life [4, 5]. These findings emphasize the need for a unifying definition and consensus about palliative rehabilitation in cancer in future official documents. While participants were able to deduce the concepts of care, translating it into routine patient care seemed to be challenging. Bridging this gap requires concerted efforts and prioritizing multidimensional care, fostering interdisciplinary collaboration, and maintaining a patient-centred approach.

Another significant finding from this study is the ambiguous distinction between palliative rehabilitation and traditional palliative care, which was evident across all sub-studies. Professionals in the field exhibited marked differences in their placement of palliative rehabilitation within the cancer trajectory, and sometimes even considered it equivalent to existing palliative care. This lack of clarity is unsurprising, given that palliative rehabilitation in cancer aligns with contemporary definitions of palliative care [29]. Moreover, cancer rehabilitation has historically been associated with curative cancer treatment and the care of cancer patients before, during and after treatment—a domain that continues to evolve [30]. Consequently, clarifying the concepts of palliative rehabilitation in cancer becomes essential for its successful integration into clinical practice.

The overall findings from this study also reveal that integration of palliative rehabilitation in cancer into clinical practice encounters several common barriers. These include insufficient funding, a lack of well-defined care pathways, and lack of expertise among healthcare professionals. The same barriers have also been acknowledged in the WHO policy [12]. They suggest evidence-based solutions for better integration of palliative rehabilitation including adoption into policy and guidelines, health planning and funding health professional training curriculums and models for care delivery.

A further significant finding, which emerged solely from the interviews, pertains to the inclusion of relatives in care. While most stakeholders emphasized the importance of involving family members as carers supporting the patient, only a few stakeholders from Norway, Denmark, and Italy explicitly addressed the need to focus on family members’ own needs. This perspective aligns with the WHO policy brief, which emphasizes that palliative rehabilitation should consider both the patient’s and the family’s needs and quality of life [12]. Although the suggested interventions often revolve around educating and assisting relatives, it is crucial to recognize the stress and challenges faced by these family members in such situations. Developing strategies to support their needs is essential for their long-term wellbeing [31, 32].

### Study strengths and limitations

A notable strength of this study lies in its utilization of mixed methods, combining results from three sub-studies. This approach facilitates an exploration of different aspects of the research questions, providing a more comprehensive understanding of the phenomena under study, in this case current integration of palliative rehabilitation in cancer. Using both quantitative and qualitative data, the study maximizes the strengths of each data type while mitigating the limitations inherent in any single approach [21]. By analysing the data separately and subsequently synthesising them, allow us to cross-verify the findings in a joint display, yielding insights that would not have emerged from considering only one method. Overall, this rigorous methodology contributes to the study’s overall quality, bolstering its validity and reliability.

However, some limitations remain. Conducting mixed-method research is more complex than using a single method. To address this challenge, we composed a research team with expertise and experience in both qualitative, quantitative, and mixed methods. Additionally, collecting data from multiple countries introduces logistical challenges, including language barriers, cultural differences, and variations in healthcare systems. These factors may impact the consistency of data collection. Given the scarcity of official documents that included the concept of palliative rehabilitation, and the interview findings indicating its limited use in clinical practice, it is a potential limitation that informants may have responded to the questions based on a palliative care context.

Not all documents analysed were in English or a Nordic language, whereby we had to rely on the translated extractions provided by authors from France and Italy. Similarly, stakeholder interviews, except those conducted in Norwegian and Danish, were carried out in English by non-native but fluent English-speaking researchers and stakeholders. As a result, some nuances may have been lost or difficult to express. However, we aimed to minimize this threat to validity by using open-ended questions, simple and clear language, paraphrasing and summarizing answer to confirm understanding, and regularly checking for comprehension. Further, the study interviewed only 4–6 respondents in each of the five countries, and the uneven distribution of survey responses (e.g., Italy versus Denmark) could limit the transferability of findings.

Additionally, the selection of documents for the document analysis, interviewees for the stakeholder interviews and respondents for the online survey were conducted by the lead investigators in the partnership countries. Though they received guidance on sample selection for all three sub-studies, the samples are convenience samples that may not be representative and could limit the generalisability of the results. This limitation is further heightened by the fact that we do not have information on response rates for the online survey, nor for the stakeholder interviews.

Furthermore, when synthesizing results from three distinct data sets, one strand of data may unintentionally carry more weight than another, potentially leading to conflicting interpretations. To mitigate this, we engaged in thorough discussions of the mixed results until consensus was reached. We then presented the findings from the three sub-studies as evenly as possible, enhancing the trustworthiness of our results. Finally, the findings represent a snapshot of the current situation, thus the results may not be widely representative.

### Implications for practice and further research

As the concept of palliative rehabilitation in cancer is evolving, our findings have demonstrated that it lacks clarity and is not widely understood or used. As this field develops, it would be paramount to address the perceived blurred distinction between palliative rehabilitation and traditional palliative care by working towards a concerted definition of the concept. Though the results showed that integrated care has been achieved in some oncology- and palliative care services in two countries, there is a need for a robust evidence synthesis to support wide-spread adoption of palliative rehabilitation in cancer care.

Evidence of effectiveness to justify recommendations of integration of palliative rehabilitation in cancer is emerging, but more research is needed, especially regarding different models of palliative rehabilitation [15–17]. One such model will be examined through the previously described multinational randomised controlled trial INSPIRE [19].

The success of a potential implementation process, based on this evidence, across European countries, would benefit from an agreed framework for practice and policy specific to palliative rehabilitation in cancer. This is planned in the INSPIRE programme through a formal consensus process with experts across rehabilitation, oncology, and palliative care [19].

## Conclusion

Our findings from this mixed-method multinational study highlight the limited integration of palliative rehabilitation in cancer care both within official documents and in clinical practice across five INSPIRE partnership countries. Integrated palliative rehabilitation services were siloed within a few institutions in secondary healthcare systems in Denmark and the UK. More widespread adoption of palliative rehabilitation in cancer remains restricted by lack of an agreed definition, insufficient funding, scarcity of care pathways that combine rehabilitation and palliative care, and inadequate expertise among healthcare professionals. Addressing these challenges will help ensure continuity of care for people with incurable cancer, with interventions that align with individuals’ needs and/or goals. Through INSPIRE we aim to progress this agenda.

## Electronic Supplementary Material

Below is the link to the electronic supplementary material.

Supplementary Material 1

Supplementary Material 2

Supplementary Material 3

Supplementary Material 4

Supplementary Material 5

## Data Availability

All data supporting the findings of this study are available within the paper and its Supplementary Information. Additional enquiry regarding the data availability can be directed to the corresponding author upon reasonable request.
