# Supplementary material for: Integration of palliative rehabilitation in cancer care: a multinational mixed method study
Source: BMC Palliat Care. 2024 Nov 18;23:267. doi: 10.1186/s12904-024-01586-1 (PMC11572245; doi:10.1186/s12904-024-01586-1)
Supplement: Supplementary file 2 — Supplementary Material 2 [file 12904_2024_1586_MOESM2_ESM.pdf]

WP 1 – part 3: In–depth interviews of stakeholders in the EU project INSPIRE – Semi-structured Interview guide

Informants: Four stakeholders from each of the participating countries in the INSPIRE intervention: Norway, Denmark, UK, France, and Italy.

Introduction:

- Thank you for your willingness to participate in this stakeholder interview in the EU-project INSPIRE

About the INSPIRE project and this interview:

- INSPIRE stands for INtegrated Short-term PallIative REhabilitation to improve quality of life and equitable care access in incurable cancer.
- The overall objective of INSPIRE project is to test the clinical and cost effectiveness of an integrated short-term palliative rehabilitation intervention to improve function and quality of life in people affected by incurable cancer and reduce the burden of care for their families.
- However, the project includes eight different work packages, and is a collaboration between the six countries: Norway, Denmark, UK, France, Belgium, and Italy.
- In Norway, at Center for Crisis Psychology and University of Bergen, we are responsible for WP 1 aiming to compare models and levels of integration between rehabilitation, oncology, and palliative care services across different healthcare systems in Europe.
- This interview is one part of this WP, where we are exploring stakeholders' experiences of how palliative cancer rehabilitation is integrated in clinical practice in the respective countries

How the interview will be conducted:

- The interview will be conducted as a conversation between you and me (the researcher).
- I have some key questions to ask about your experiences of and views on how palliative cancer rehabilitation is integrated in clinical practice in your country.
- Preferably, I want you to talk about your experiences about the topic as comprehensively as possible, whereby I come in with my questions as we go along.
- No answers are right or wrong: we want to get your experiences and views about the topic.

- The interview will last approximately one hour – depending on how much there is to share.

About the Data management:

- This interview will be audiotaped on Zoom.
- After the interview the recording will be transcribed verbatim, before it is deleted
- No identifying information, except from land of origin - will be transcribed (e.g., names, geography, organization etc.)

Sociodemographic variables

- Before we start the recording, I need some background information
- Country, age, gender, profession, title, workplace (hospital, primary healthcare, hospice, etc), years of work experience

Interview Number	Country	Gender	Age	Profession	Title	Workplace	Years of experience in oncology/ palliation

- Do you have any questions about the project or the interview before we start?

The interview and recording:

Today's interview is with participant number xx.<br>• First, I need you to confirm that you have read the information letter and give your verbal consent to participate in this study as described in the letter?	Start the recoding
- As I explained, this interview is about your experiences of how palliative cancer rehabilitation is integrated in clinical practice in your country. (Go to main questions)	
**Main Question**	**Follow – up questions if necessary**
1. First, can you please tell me how you would define or your understanding of the concept palliative rehabilitation?	The aim of palliative rehabilitation?<br><br>Rehabilitation as an essential service towards the end of life?
2. WHO's policy on Universal Health Coverage states that both rehabilitation and palliative care are essential quality health services.<br><br>In what way do you think this is the case for your country?	Why/why not?
3. Can you please tell me about your experiences or your views on how palliative cancer rehabilitation is integrated in clinical practice in your country	- Level of integration: between primary, secondary, and tertiary health systems?<br>- Profession: single or multidisciplinary approach?<br>- Common interventions?<br>- Who are funding these services?
4. How will you describe the access to palliative care services contra rehabilitation services for cancer patients with incurable cancer in your country?	- Contra other groups e.g., cardiac, stroke, respiratory diseases?
5. What do you think would be the best way to deliver palliative rehabilitation in the future?	- In one or across settings?<br>- As a single or parallel approach to oncology and palliative care?
6. Now, I have been through all my questions. Is there anything you would like to add or elaborate on?	-
- Then, I thank you very much for your time and important input into this topic – and I will stop the recorder.	- Stop the recorder

After the interview:

- Thank you so much for participating in this interview. It is very important for us to interview a stakeholder with your expertise, and it is much appreciated.
- How did you experience the interview?
- Repeat information about safe handling of data.
- Do you have any questions or comments before we say goodbye?
