# Supplementary material for: Integration of palliative rehabilitation in cancer care: a multinational mixed method study
Source: BMC Palliat Care. 2024 Nov 18;23:267. doi: 10.1186/s12904-024-01586-1 (PMC11572245; doi:10.1186/s12904-024-01586-1)
Supplement: Supplementary file 3 — Supplementary Material 3 [file 12904_2024_1586_MOESM3_ESM.pdf]

Invitation to take part in the research project
INSPIRE: **I**ntegrated **S**hort-term **P**all**I**ative **RE**habilitation to improve quality of life and equitable care access in incurable cancer

This is a EU-funded project that will investigate a short-term palliative rehabilitation intervention for people with incurable cancer through a randomised controlled trial (RCT) in five European countries. Palliative rehabilitation empowers people with incurable conditions to actively manage their condition, enabling them to live fully and enjoy the best quality of life possible.

Prior to start-up of the RCT in the autumn 2023, we wish to gain insight into variations in practice within the partnership trial sites. We hope that you are willing to contribute by completing a short questionnaire that can be answered by ticking boxes and should take no longer than 10 minutes. We will not collect any personal data from you and your responses will be completely anonymous.

The questionnaire should work fine with any internet browser, but in case it does not display properly, please note that the platform we are using - SurveyXact - is optimised for the latest versions of the browsers Google Chrome, Firefox and Safari.

Please complete the questionnaire within 31st May 2023.
Thank you very much for your help - it is much appreciated!

You have the option to read more information about the survey and data handling before deciding whether to take part.

☐ I consent to taking part and to the use of my data and I am happy to complete the survey without further information

☐ Ticking this box will direct you to a page with further information about the survey and data handling

Participant information sheet

Purpose of the project

This survey is part of the research project "INSPIRE: INtegrated Short-term PallIative REhabilitation to improve quality of life and equitable care access in incurable cancer". At the core of the project is a randomised controlled trial to assess the clinical and cost effectiveness of palliative rehabilitation on quality of life, disability, symptom

burden and goal attainment in people with incurable cancer. The trial will recruit participants across five European countries (UK, France, Italy, Denmark, and Norway).
In this part of the project, we wish to understand how palliative rehabilitation is currently integrated across oncology and palliative care services in Europe. We are doing this by conducting a comparative analysis in the participating countries where one element is an online survey with medical, health care and allied health professionals. It is with respect to this survey that you are invited to take part.

Who are responsible for the research project?

The project is funded through Horizon Europe and coordinated from France by Dr Guillaume Economos at Hospices Civils De Lyon. Professor Matthew Maddocks and Dr Joanne Bayly at King's College London, UK are responsible for the scientific and project management aspects respectively. A research group at the Centre for Crisis Psychology, University of Bergen, Norway, is responsible for the work package concerning the comparative analysis, including this survey. The group is led by Professor Line Oldervoll, with team members Professor May Hauken, Associate Professor Guro Birgitte Stene, Postdoctoral Researcher Hilde Hjelmeland Ahmedzai, and Research Assistants Constance Storvestre and Skjalg Eirik Vervik.

Why have you been asked to participate and what will it involve?

Nine hospitals/hospice/cancer centres across the five collaborating countries will participate in the recruitment of patients into the randomised controlled trial. In preparation for the trial, we wish to gain insight into variations in practice, referral criteria and patient pathways within the partnership trial sites. We do this by gathering information from professionals working in clinical care within oncology, palliative care and rehabilitation at these hospitals/cancer centres. We have sent an email invitation with a link to the survey to the INSPIRE Lead for your country, with a request to forward that on to applicable medical, health care and allied health professionals within the trial sites. That is why you have received the invitation, and we hope that you are willing to contribute by completing a short questionnaire (mainly ticking boxes) that should take no longer than 10 minutes.

How is your data collected and handled?

The survey tool used for gathering the information is called SurveyXact, a leading survey tool in Scandinavia, developed by the Danish company Rambøll. Data security is their highest priority and SurveyXact fully complies with the EU's General Data Protection Regulation that came into force in 2018. GDPR for questionnaire surveys (surveyxact.com)

In this survey we do not collect any personal data (such as name, email address etc.) and completion of the survey is completely anonymous. Respondents' IP addresses are anonymized, so neither we (members of the research team) nor employees at SurveyXact, have access to view or use the IP addresses used to complete the survey. Thus, you cannot be identified by completing the survey. Based on this, the survey does not require approval from ethical or research governance body, nor data protection authority. The data will be processed in accordance with the General Data Protection Regulation (GDPR). The anonymous data will be transferred from the survey tool to a database containing all data from all survey respondents and stored at a safe server at the University of Bergen. The analysed data will be used to inform the research of the INSPIRE project, specifically in preparation for the trial that will start in the autumn 2023. The results from the survey will be published in a report to the Consortium members of the INSPIRE research project and will further be part of the overall project report for EU Horizon. The anonymous data may also be part of research publications for the comparative analysis.

Where can I find out more?

If you have questions about the survey part of the project, please contact:

- Hilde Hjelmeland Ahmedzai, PhD, Survey co-ordinator, email: hilde.ahmedzai@uib.no Centre for Crisis Psychology at University of Bergen or
- Professor Line Oldervoll, Lead for INSPIRE Work Package 1, email: Line.Oldervoll@uib.no
- Janecke Helene Veim, Data Protection Officer at the University of Bergen, email: janecke.veim@uib.no
- Ivan Dalsgaard Sørensen, Data Protection Advisor at Rambøll Management Consulting (for SurveyXact), email: ivds@ramboll.com

Participation is voluntary

Participation is voluntary and you can opt out by not continuing to the questions and close the web browser. If you choose to take part in the survey, please tick the box stating that you have received information and consent to participate. We also ask if we may use the responses you have provided if you have completed some questions but not all of them.

Thank you for your interest – it is much appreciated!

Yours sincerely,

Hilde Hjelmeland Ahmedzai, PhD

Survey Co-ordinator
in Norway

Professor Line Oldervoll

Leader of the INSPIRE project

I have read information about the INSPIRE project and use of data and consent to participate

☐ I consent to participate and to the use of data as described in the participant information sheet above

If I do not respond to all questions or press "Finish" on the last page, I consent to the use of data that has already been entered by me

☐ I consent to the use of data already entered

Thank you and please press "Next" to start the survey.

You can close the browser (web page) if you decide not to take part in the survey

Please pick the name of the institution where you work

☐ Guy's and St Thomas' Hospitals NHS Foundation Trust, UK

☐ King's College Hospital NHS Foundation Trust, UK

☐ Institute of Genetics and Cancer/Edinburgh Cancer Centre (UK)

☐ St Columba's Hospice Care (UK)

- ❑ Hôpital Lyon Sud-Hospices Civils de Lyon (France)
- ❑ Fondazione IRCCS Instituto Nazionale dei Tumori (Italy)
- ❑ Azienda Unità Sanitaria Locale-IRCCS-Reggio Emilia Italy)
- ❑ Copenhagen University Hospital (Denmark)
- ❑ St. 0lavs University Hospital (Norway)
- ❑ Other, please specify__________

Professional background

- ❑ Medical
- ❑ Nursing
- ❑ Physiotherapy
- ❑ Occupational therapy
- ❑ Dietitian/Nutrition
- ❑ Social work
- ❑ Psychology
- ❑ Speech therapy
- ❑ Spiritual care
- ❑ Complementary care
- ❑ Other, please specify__________

Where do you work?

- ❑ Department of oncology
- ❑ Palliative care in-patient unit
- ❑ Palliative care hospital consult service
- ❑ Rehabilitation or Therapies service
- ❑ Department of physiotherapy/occupational therapy
- ❑ Hospice
- ❑ Other, please state__________

In your work with cancer patients, are you part of a multiprofessional/multidisciplinary team (MDT)?

- ❑ Yes
- ❑ No
- ❑ Neither Yes or No, we are structured in a different way, please explain below

Which professions are part of the MDT? (If you are part of more than one MDT, please reply with regard to the MDT discussing patients with incurable cancer)

Please tick all that apply.

- ❑ Oncologist(s)
- ❑ Palliative Medicine Consultant(s)/Specialist(s)

- [ ] Haematologist(s)
- [ ] Radiologist(s)
- [ ] Surgeon(s)
- [ ] Physical Medicine Physician(s)/Physiatrist(s)
- [ ] Oncology Nurse(s)
- [ ] Palliative Care Nurse(s)
- [ ] Physiotherapist
- [ ] Occupational therapist
- [ ] Dietitian(s)/Nutritionist(s)
- [ ] Social Worker(s)
- [ ] Psychologist(s)
- [ ] Spiritual, religious and pastoral care representative
- [ ] Other, please specify__________

What is the frequency of the MDT meetings?

- [ ] Daily
- [ ] Weekly
- [ ] Every 2 weeks
- [ ] Monthly
- [ ] Other, please specify__________

Do you follow guidelines/protocols/standardised care pathways (SCP) on the management/follow-up care of cancer patients?

- [ ] Yes
- [ ] No
- [ ] Don't know

Is palliative care a component of the cancer care guideline/protocol/care pathways?

- [ ] Yes
- [ ] No
- [ ] Don't know

Is rehabilitation a component of the cancer care guideline/protocol/care pathways?

- [ ] Yes
- [ ] No
- [ ] Don't know

Are these guidelines local, regional or national?

- [ ] Local
- [ ] Regional
- [ ] National
- [ ] Don't know

Are patients with incurable cancer routinely screened/assessed for rehabilitation needs in your workplace?

- ❑ Yes
- ❑ No
- ❑ Don't know

If not routinely screened, what are triggering factors for referral to palliative rehabilitation?

- ❑ Poor physical functioning
- ❑ Planned discharge from hospital to home
- ❑ On patient request
- ❑ On request from carers
- ❑ In line with clinical guidelines/care pathways
- ❑ We do not refer to palliative rehabilitation
- ❑ Other, please specify__________

What are the point(s) of contact between cancer palliative care and rehabilitation services?

Plese tick all that apply.

- ❑ Joint multidisciplinary team meetings
- ❑ Other regular collaborative meetings
- ❑ Ad hoc meetings according to patients' needs
- ❑ One-to-one phone calls to discuss individual patients
- ❑ Members of the rehabilitation team/services screen palliative cancer patients on the ward
- ❑ Joint educational activities
- ❑ Management meetings
- ❑ No organised/regular contact
- ❑ Other, please specify__________

Are electronic patient records shared between department/services?

- ❑ No
- ❑ Yes, between oncology and palliative care
- ❑ Yes, between palliative care and in-patient rehabilitation services
- ❑ Yes, between all departments/units in the hospital
- ❑ Yes, between all levels of care (primary, secondary and tertiary)
- ❑ Other, please specify__________

Does the IT patient platform work satisfactorily? (with regard to facilitating collaboration and integration between professions, departments and levels of care)

- ❑ Yes
- ❑ No
- ❑ Don't know
- ❑ Not applicable

Are there regular multiprofessional educational activities in your department?

(I.e. jointly for doctors, nurses, physiotherapists, occupational therapists, dietitians etc)

- ☐ Yes
- ☐ No
- ☐ Don't know

Which cancer palliative rehabilitation services are you aware of that are available for patients with incurable cancer within the catchment area of your hospital?

Plese tick all that apply.

- ☐ Dedicated cancer palliative rehabilitation service(s) at the hospital
- ☐ Dedicated cancer palliative rehabilitation service(s) in primary care
- ☐ Dedicated cancer palliative rehabilitation service(s) in hospice(s)
- ☐ No dedicated palliative rehabilitation service - rehabilitation for patients with incurable cancer is offered within generic cancer rehabilitation service(s)
- ☐ Not aware of any service offering rehabilitation for patients with incurable cancer
- ☐ Other, please specify__________

To your knowledge, what are the rehabilitation components available for patients with incurable cancer in the services they can be referred to?

Please tick all that apply.

- ☐ Individual goal-oriented rehabilitation
- ☐ Physical exercise/activity
- ☐ Physiotherapy
- ☐ Psychoeducation
- ☐ Occupational therapy
- ☐ Nutritional advice/support
- ☐ Psychological counselling
- ☐ Speech therapy
- ☐ Peer support
- ☐ Involvement of carers
- ☐ Don't know
- ☐ Other, please specify__________

To your knowledge, where is palliative care delivered in your city/local area?

Please tick all that apply

- ☐ Palliative care in-patient unit(s)/department
- ☐ Palliative care hospital consult team(s)
- ☐ Palliative care out-patient clinic(s)
- ☐ Community-based or home palliative care
- ☐ Hospice(s)
- ☐ Nursing homes (and/or homes for the elderly)
- ☐ Don't know
- ☐ Other, please specify: __________

What is your view on the following statements?

	Strongly agree	Agree	Neither agree or disagree	Disagree	Strongly disagree	Don't know/cannot decide
Palliative care and palliative rehabilitation offer the same services	☐	☐	☐	☐	☐	☐

Palliative care focuses mainly on symptom palliation	☐	☐	☐	☐	☐	☐
Palliative rehabilitation focuses mainly on physical function	☐	☐	☐	☐	☐	☐
Rehabilitation is most appropriate for curative cancer patients	☐	☐	☐	☐	☐	☐
Rehabilitation is not appropriate for cancer patients towards the end of life	☐	☐	☐	☐	☐	☐

For patients with incurable cancer in your hospital, how do you rate the continuity of care between the hospital and primary care?
0 = Poorly and 100 = Excellent

____

Please state below if you have any other comment relating to palliative cancer rehabilitation

Please state below if you have any other comment relating to the topic(s) of this survey

Thank you for your time and contribution! Please press "Finish" to submit the questionnaire (This will direct you to the INSPIRE project website where you can read more about the project or just close the browser window)
