# Supplementary material for: Integration of palliative rehabilitation in cancer care: a multinational mixed method study
Source: BMC Palliat Care. 2024 Nov 18;23:267. doi: 10.1186/s12904-024-01586-1 (PMC11572245; doi:10.1186/s12904-024-01586-1)
Supplement: Supplementary file 4 — Supplementary Material 4 [file 12904_2024_1586_MOESM4_ESM.pdf]

Publisher, year	Type and objective	Main themes from extraction and analysis of documents				
		Needs and access to rehabilitation	Settings, responsible parties and essential services	Timing and care pathways	Goals, interventions, and professionals	Research and future PR
**Danish Health Authority (2018)[1]** .	Policy - to outline the responsibilities of the municipalities regarding rehabilitation	Rehabilitation is used extensively throughout document but not in context of cancer or palliative care in cancer.				
**Danish Health Authority (2018)[2]**	Care pathway - To describe recommended care pathways for rehabilitation and palliative care for cancer.	People living with cancer in all phases of the disease trajectory.	Main responsibility for R is within community health service while PC depends on what service is required.<br>R and PC should run in parallel and/or continuously within hospital and community care.	R and PC is described as two separate care pathways<br>R and PC should be initiated as early as possible after diagnosis and monitored throughout disease trajectory.<br>Initiation of R and PC should be based on the individuals needs of patients.	R and PC have a large overlap in terms of professional content, often different goals. i.e. R aims to promote function and independence, while PC aims to reduce physical, psychological, social and spiritual suffering of patients and their relatives. Patients might need both R and PC services although they have different goals.<br>Organisations and health professionals should have the necessary competence and skills to provide R and PC to meet patient's needs.	The overlap argues for a high degree of integration between the two care pathways.
**Rehabilitation Forum Denmark (2022)[3]**	White paper - To provide a status of rehabilitation services and current challenges provided by and to be used by all stakeholders.	People living with incurable diseases, including cancer.	Should be integrated into the municipal rehabilitation services to improve continuity of care and meet needs of patients.	R and PC typically represent separate care pathways as they focus on different principles.<br>Differences are lessened if R (improving function, coping and QoL is offered later and PC (symptom relief, QoL, and preparing for death) earlier in the disease trajectory.	Defines rehabilitation according to WHO. In this context, palliative rehabilitation (PR) is a new term introduced as rehabilitation for people with advanced cancer aiming to reduce complications and promote coping of symptoms. A synonym to PR is the term "rehabilitative palliation" which stems from palliative care.<br>No formalized descriptions of competences needed by rehabilitation specialist are available.	PC and R represent two different fields of research and clinical practice; however, they should be integrated into care pathways for people with advanced diseases.
**Danish Cancer Society (2021)[4]**	Report - To monitor the quality of rehabilitation services provided to cancer patients.	People living with cancer who need rehabilitation and palliative care services.	Not described	R and PC might follow different referral pathways.<br>Referral is primarily after completion of treatment; however early initiation does occur	R and PC are overlapping in terms of organisation, professional content, and timing.<br>Both R and PC have similar goals i.e., supporting patients and their carers to cope with the situation.	Not described
**Haute Autorité de Santé (2002)[5]**	Guideline -These guidelines aim to guarantee the right to and the access to palliative care.	People who need palliative care services	Not described	Not described	Patients with dysphagia should receive speech and language therapy rehabilitation.<br>PTs and OT should be part of the MDT to meet patients need for rehabilitation.	Not described

**Direction Générale de l'Offre de Soins (2023)[6]**	Strategy - This document is a policy guiding the further development and structuration of the palliative care offer across the state between 2024 and 2034	People who need palliative care services	Palliative day hospital	A care plan can include rehabilitation as a one-shot intervention or as part of a longer follow up.	Rehabilitation practitioners must be part of an MDT providing palliative care.	Not described
**Association française des Soins Oncologiques de Support (2013, 2018)[7]**	Guideline - To promote physical activity and rehabilitation in breast cancers all along the disease trajectory.	States that as according to National Cancer Plans I and II, rehabilitation is recommended for breast cancer patients, but document do not describe this in context of advanced cancer or in a palliative care setting.				
**National Cancer Institute (2021)[8]**	Guideline - To define at a national level the organizational principles related to the implementation of the support care pathway for patients with cancer	Rehabilitation functional assessment is recommended as part of the supportive care in cancer to start “adapted physical activity” but recommendation applies only to patients undergoing treatments or being cured				
**Haute Autorité de Santé (2019)[9]**	Guideline - To guide the promotion, consultation and prescription of physical activity and sport for health, including specific guidance for people with three most common cancers (breast, colon and prostate).	Focus on the role of physical activity, not specific to palliative context, expect for in the case of risk of bone fractures.				
**National Cancer Institute (2017)[10]**	Guideline - To summarize the data on physical activity benefits in cancer and provide guidelines on the integration of physical activity during and after cancer treatments	Focus on physical activity during and after cancer treatments, not specific to palliative care context				
**Italian Association of Medical Oncology/ Italian Society of Palliative Care (2015)[11]**	Other (consensus) - To guide healthcare professionals to offer the most appropriate treatment pathway for patients with advanced cancer.	People with advanced cancer with complex needs and where there are no effective therapies.	In the home	All stages of the cancer disease.	R is mentioned to argument in favor for the need to have a comprehensive approach in the treatment of cancer including attention to complex needs and preserving QoL in all its components.<br>“Unit of Palliative Care at Home” is an integrated complex of professional medical, nursing, rehabilitative, and psychological health care services, as well as social and tutelary services and spiritual support.	Not described

**Permanent Conference for relations between the State, the Regions and the autonomous Provinces of Trento and Bolzano 2019)[12]**	Policy Directive - To revise the organizational guidelines and recommendations for the Oncology Network that integrates acute and post-acute hospital activity for the promotion and improvement of the quality, safety and appropriateness of supportive interventions.	Cancer patients with complex needs, including patients at the terminal stage of life (e.g., at hospice).	Palliative home care, provided by a specific unit (UCP) at a specialist (hospital) and residential health and social care (hospice) level.	R and PC are essential part in the oncological treatment pathway and should be initiated during hospitalisation and modulated throughout the course of the disease.	R is foreseen as part of PC.	Not described
**Italian Ministry of Health (2019)[13]**	Official statement or declaration - To summaries the goals achieved and the critical issues that have emerged in the process of implementation and development of assistance networks in palliative care and pain therapy for adults and children, eight years after the entry into force of Law No. 38 of 15 March 2010	Children and adults in need of palliative care and pain therapy	Not described	Not described	The terms ‘rehabilitative’ and ‘rehabilitation’ are listed as expected competencies in PC and pain management. The PTs are professionals who contributes in a fundamental way to PC together with the nurse, the dietician, the social worker, the social-health worker and the psychologist.	Not described
**Italian Ministry of Health (2021)[14]**	Policy - To ensure appropriate qualified palliative care and pain therapy for patient and his family for the period 2010-2020.	Patients and families in need of PC and pain therapy	Not described	Not described	PC in hospice and at home is described as an integrated complex of professional medical, nursing, rehabilitative and psychological health care services, as well as social, tutelary and hotel services, and spiritual support. Access to R is essential part of PC services and pain therapy facilities in the end-of-life. RPs are listed as practitioners who are part of the qualifying standards for PC services.	Not described
**Ministry of Health and Care Services in Norway (2018)[15]**	Strategy - To provide directives for cancer care in Norway for the period 2018-2022	Patients receiving life-prolonging- and palliative care	Both in the specialist - and community health service<br>.	R and PC should be offered as an integrated part of care pathway but described as separate pathways.<br>PC should be integrated early during cancer disease to achieve better coping.	Patients receiving life -prolonging and palliative care needs rehabilitative measures to increase QoL, reduce symptoms and cope with life situation.<br>The PC teams are interdisciplinary, and often consists of doctor and nurse, with the assistance of a physiotherapist, occupational therapist, social worker, psychologist/ psychiatrist, and priest.<br>The Vardesenter is an arena for people affected by cancer and their relatives with activities that promote quality of life, well-being, and coping.	Many patients can live good lives even though they have cancer with spread, and therefore need life extension treatment, rehabilitation, learning and coping services, palliative treatment, nursing, and care.
**Norwegian Cancer Society**	Strategy - To work to prevent and fighting cancer as well as improving the quality of life	Adress rehabilitation in context of patients with complex needs, do not refer to advanced cancer or palliative care specifically.				

**(2018)**[16]	for people with cancer and relatives					
**Norwegian Directorate of Health (2019)**[17]	Guideline - To improve the treatment of patients with incurable cancers and a limited lifespan, and to ensure an equally good treatment offer throughout the country	Patients with cancer who needs PC	Hospitals	PC should be initiated early in disease trajectory.	Focus on the assessment of physical function as a key aspect of PC (symptom management) and starting point for R.<br><br>Describes how patients can have symptoms of physical, psychosocial and spiritual character; loss of physical function is a key characteristic; pt often suffer from late effects that impair their quality of life, need for care beyond curative treatments;(the term rehabilitation not used) but describe how this care should be about the pt preferences, how pt wish to spend his time, what type of treatment or care he will prioritize; patient perspective should be the focus through "Advance Care Planning"	Not described
**Norwegian Directorate of Health (2017)**[18]	Care Pathway - To ensure safe and proper conditions for those who have received a cancer diagnosis and that the need for follow-up beyond the cancer treatment	All patients with cancer regardless of curative or palliative intention.	Both specialist -and the community health service (however responsibility is not explicitly described)	Three interaction points for dialog: at diagnosis, 3-4 months post diagnosis and 12-18 months post diagnosis	R is listed as one of themes that should be individualised and included in the assessment of needs of the patients.<br>The dialog should preferably be conducted by a doctor or nurse, or other health personnel, include information to patients about available sources of help and support, and finally, the result of the dialog should be documented in patient journal and shared with primary health service.	R is listed as one of the key areas to be targeted by the Care Pathway at Home.
**NHS England (2019)**[19]	Strategy - To detail what is required by the National health Service towards 2029	R is not used to describe care in relation to cancer.				
**NHS England (2018)**[20]	Guideline - To provide practical advice and case studies to support Allied Health Professionals in Action.	Patients with cancer	Not described	Patients with metastatic disease often do not see AHPs until the final few months of life.	Specialist R is described as particularly important for those with complex care needs. AHPs understand these complex needs and can redesign services around them, creating links between primary and secondary care, across specialties and across health, public health, and social care. R interventions are recommended for patients with painful bone metastases, to improve quality of life.	Research demonstrated need for input from RPs in the MDT approach to patients with painful bone metastases. Potential for earlier preventive and rehabilitation interventions.
**The Scottish Government (2008)**[21]	Strategy - To set out the specific challenges for cancer care in Scotland as well as strategies for improvement	R is mentioned in relation to cancer and managing cancer symptoms, but not in relation to PC.				

**National Institute for Clinical Excellence (2004)[22]**	Guideline - To guide how to improve the quality of supportive and palliative care for cancer patients	All cancer patients Variable access to R applies to all cancer patients in all care locations. R access is currently expanding to more tumour groups, including people whose disease is progressively deteriorating	R should be given alongside specialist PC services and might have significant interactions with them. Specialist PC services in hospitals, hospices and primary care should form close working relationships with RPs.	All patients are likely to need R at some point in the disease pathway. Supportive care, which includes R, should be given equal priority with other aspects of care and be fully integrated with diagnosis and treatment.	R attempts to maximise patients' ability to function, to promote their independence and to help them to adapt to their condition. R offers a major route to improving their QoL, no matter how long or short the timescale. It aims to maximise dignity and reduce the extent to which cancer interferes with an individual's physical, psycho-social, and economic functioning. The importance of R in improving patients' lives is gaining more recognition and they are increasingly being seen as integral to patient care. Specialist PC teams should have access to the full portfolio of supportive care services, including R. Assessments should encompass all aspects of supportive and PC including the preferences of patients and carers with respect to R. R is listed as one of the areas which will need specialist staff to meet patients' needs. High quality R should be ensured through establishing 'clinical specialist' or 'consultant therapist' posts across all AHP groups in cancer and PC.AHP should be part of MDT meetings as appropriate.	Access to R needs to be considered in advancing disease or those requiring support at the end of life. Clinicians and RPs express concern that needs are not meet (e.g., due to lack of knowledge of what skilled AHP can offer), formal evaluation of services is needed to meet objectives that all patients should have their needs for R assessed throughout the care pathway, including at the end of treatment and towards the end of life. Research on the impact of R interventions is lacking in UK settings.
**NHS England (2022)[23]**	Strategy - To provide strategic direction to AHPs across England in the period 2022-2027 and help AHPs maximize their contribution to improve health outcomes for all, provide better quality care, and improve sustainability of health and care services	No mention of palliative care, other than a link to document 19.				

[1] Danish Health Authority (2018): Guideline on rehabilitation and functional level in municipalities and regions.
[2] Danish Health Authority (2018): Care pathways for rehabilitation and palliative care in connection with cancer.
[3] Rehabilitation Forum Denmark (2022): White book about rehabilitation.
[4] Danish Cancer Society (2021): Report about rehabilitation and palliative care in connection with cancer.
[5] Haute Autorité de Santé (2002) : Modalités de prise en charge de l'adulte nécessitant des soins palliatifs.
[6] Direction Générale de l'Offre de Soins (2023) : INSTRUCTION INTERMINISTÉRIELLE N° DGOS/R4/DGS/DGCS/2023/76 du 21 juin 2023 relative à la poursuite de la structuration des filières territoriales de soins palliatifs dans la perspective de la stratégie décennale 2024-2034.
[7] Association française des Soins Oncologiques de Support (2013, 2018) : Référentiels inter-régionaux en Soins Oncologiques de Support - Activité Physique adaptée, rééducation et cancer du sein.
[8] National Cancer Institute (2021) : Référentiel organisationnel National Soins Oncologiques de support des patients adultes atteints de cancers.
[9] Haute Autorité de Santé (2019) : Prescription d'activité physique et sportive Cancers - sein, colorectal, prostate.
[10] National Cancer Institute (2017): Bénéfices de l'activité physique pendant et après cancer des connaissances scientifiques aux repères pratiques.
[11] Italian Association of Medical Oncology/ Italian Society of Palliative Care (2015): Early and simultaneous palliative care.
[12] Permanent Conference for relations between the State, the Regions and the autonomous Provinces of Trento and Bolzano 2019): Agreement between the Italian State and the Regions pursuant to article 4 of Legislative Decree 28/08/1997 on the document "Review of the organizational Guidelines and recommendations for the Oncology network which integrates the hospital activity for acute and post-acute territorial"
[13] Italian Ministry of Health (2019): Report to Parliament on the state of implementation of the Law n. 38 of 15 March 2010 "Provisions to guarantee access to palliative care and to pain management".
[14] Italian Ministry of Health (2021): Law No. 38 of 15 March 2010 and implementing measures. Regulatory collection.
[15] Ministry of Health and Care Services in Norway (2018): Living with cancer. National Cancer Strategy 2018-2022.

[16] Norwegian Cancer Society (2018): Strategy 2020-2023.
[17] Norwegian Directorate of Health (2019): Program of Action for Palliative Care in Norway.
[18] Norwegian Directorate of Health (2017): Care pathway home for patients with cancer.
[19] NHS England (2019): The NHS Long Term Plan Version 1.2 with corrections, August 2019.
[20] NHS England (2018): Quick Guide: the role of allied health professionals in supporting people to live well with and beyond cancer.
[21] The Scottish Government (2008): Better Cancer Care - an Action Plan.
[22] National Institute for Clinical Excellence (2004): Improving supportive and palliative care for adults with cancer.
[23] NHS England (2022): The AHP Strategy for England: AHPs Deliver.
