# Supplementary material for: Integration of palliative rehabilitation in cancer care: a multinational mixed method study
Source: BMC Palliat Care. 2024 Nov 18;23:267. doi: 10.1186/s12904-024-01586-1 (PMC11572245; doi:10.1186/s12904-024-01586-1)
Supplement: Supplementary file 5 — Supplementary Material 5 [file 12904_2024_1586_MOESM5_ESM.pdf]

Codes	Synthesis UK	Synthesis France	Synthesis Italia	Synthesis Norway	Synthesis Denmark	Merged synthesis
**Definition/understanding of palliative rehab (PR)**	Part of but more than PC, holistic, based on need assessment, optimizing function based on goals	PR included in PR, variable understanding from unknown to improve holistic capacity based on patients' needs.	Integrated in PC, holistic and need based approach, promote, or maintain function, including the informal careers.	Integrated but not the same as PC. Holistic and need based, optimizing function.	Integrated I PC, holistic approach, improve function	Part of PC, but not the same. Holistic approach, based on patients' needs, maintain, and promote function
**PR a used concept?**	Generally, not well known, but used in some specialized units and professions	No	Not well known and used.	No, perhaps more in some professions	Not common but used in few specialist services.	Generally, not well known or used, with exception from some specialized services and professionals in UK, Denmark and Norway
**Aim of PR**	QOL in according to goals	Improve QOL, based on what is relevant for the patient	Promote and maintain QOL, function, independence. Reduce cost for society.	QOL, optimize or maintain holistic function based on patients' perceived needs.<br>Including informal careers.	Improve and maintain QOL and function, participation in daily life	Improve/maintain QOL and function based on needs and goals. Only Norway included careers
**Common interventions**	Multidimensional interventions based on pt's needs and goals, including careers	Need based and holistic interventions	Multidimensional interventions based on patients' needs and goals. Train the caregiver.	Multidimensional interventions based on patients' needs and goals.	Different multidimensional intervention based on assessment, including assistive technology and informal careers	Multidimensional interventions based on patients' needs and goals.
**Integration**	In general, not integrated nor coordinated across levels, most work in	Some services can be defined as PR, but poorly integrated	Neither PC nor PR are well integrated, some integration of PC	Some services can be defined as PR, but mostly not well integrated.	In general, not well integrated, but PR exists	In general, not well integrated. Some services can be/are defined as PR within

	silos, also a post code lottery. PR is integrated within some services.		depending of area – north/south divide.		in some specialized units.	silos in all countries except Italy
**Professionals involved**	Multidisciplinary approach, including complementary therapy	Multidisciplinary approach	Multidisciplinary approach	Multidisciplinary approach, included outside the healthcare services (e.g. priest, NAV, school).	Multidisciplinary and holistic approach	Multidisciplinary and holistic approach. UK include complimentary therapies, and Norway also include school and social security services.
**PC and PR as essential services**	Both viewed as essential services.	Essential, but not in the same phase.	Differs regionally, north/south divide.	Considered as essential but not well developed.	PC essential service, but not PR (more in the beginning)	Bothe wide as essential services, but not in the same phase (DK og F), north/south divide (I), not well developed (N).
**Access for cancer pt (contra others)**	Varies across the country (better access for PR in bigger cities), cancer pt less access than other diseases (e.g., stroke, COPD, cardiac).	Differs around the country, less access to PR/rehabilitation than other disease (COPD, heart etc.).	Better in north and in some specialized centers.	Variers across the country – access to PR limited. Less access to rehabilitation in general than other diseases e.g., stroke.	Secret service – varies across the country. Perhaps better access than other groups because of Cancer society and other special units/services	Secret service that varies within the countries. Most countries have better access to rehab for other diseases (e.g., stroke, COPD, heart) than for incurable cancer pt.
**Patient pathways**	MC Millan pathway included PR, but not well implemented	No	PR not part of pt pathway.	No, but existing structures could facilitate PR (e.g., cancer coordinator,	Have patient pathway but rehabilitation mostly in early phase.	In general, PR is not included in pathways, except UK.

				individual plan, care pathway home)		
**Referral criteria**	Late paper referral to PC	NA	NA	NA	Varies across the country, coincidental referrals	In general, late paper referrals to PC – varying within the countries.
**Funding**	NHS and charities Funding a challenge	Social security system, dependent of priorities – disparities.	NHS, funding concerns.	NHS, varies priorities of resources both in primary and secondary healthcare system.	NHC, varies due to priorities and limited resources.	NHS with funding concerns. UK substantial charity funding for PC.
**Cost drivers**	Professionals, competing for funding, capacity, long waiting time	Not prioritized, long waiting time.	Fundings, lack of professionals and expertise.	Lack of expertise, priorities.	Lack of capacity and resources	Lack of capacity, resources, expertise, priority, waiting time
**Suggestions for future PR**	Need assessments and patients' autonomy, use of PROMS, PR included in pathways, start early, included in PC, training, PR teams in hospital/hospice with outreach	Funding, expertise, involvement from diagnose, building on existing structures, improve coordination, palliative teams referring to rehab centers.	Policy, funding, guidelines, scientific evidence, integration in PC, training professionals, telemedicine, start at hospitals and go home, build centers, develop integrated networks, equality across the country	Need og stronger focus, resources, priorities, and expertise. Use of existing structures as CCP, cancer coordinator, individual plan, networks.	Improve basic PC in municipalities, training, systematic need assessment, digital and physical follow-up, equality	Involvement from diagnoses, needs assessment, included in PC and pathways/guidelines, building on existing structures, training, resources/priorities
